# Single Nucleotide Polymorphism in ATM Gene, Cooking Oil Fumes and Lung Adenocarcinoma Susceptibility in Chinese Female Non-Smokers: A Case-Control Study

**DOI:** 10.1371/journal.pone.0096911

**Published:** 2014-05-12

**Authors:** Li Shen, Zhihua Yin, Wei Wu, Yangwu Ren, Xuelian Li, Baosen Zhou

**Affiliations:** 1 Department of Epidemiology, School of Public Health, China Medical University, Heping District, Shenyang, Liaoning Province, China; 2 Key Laboratory of Cancer Etiology and Intervention, University of Liaoning Province, China; National Health Research Institutes, Taiwan

## Abstract

**Background:**

The ataxia-telangiectasia mutated (ATM) gene plays an important role in the DNA double-strand breaks repair pathway. Single nucleotide polymorphisms (SNPs) of DNA repair genes are suspected to influence the risk of lung cancer. This study aimed to investigate the association between the ATM -111G>A (rs189037) polymorphism, environmental risk factors and the risk of lung adenocarcinoma in Chinese female non-smokers.

**Methods:**

A hospital-based case-control study of 487 lung cancer patients and 516 matched cancer-free controls was conducted. Information concerning demographic and environmental risk factors was obtained for each case and control by a trained interviewer. After informed consent was obtained, 10 ml venous blood was collected from each subject for biomarker testing. Single nucleotide polymorphism was determined by using TaqMan method.

**Results:**

This study showed that the individuals with ATM rs189037 AA genotype were at an increased risk for lung adenocarcinoma compared with those carrying the GA or GG genotype (adjusted odds ratios (OR) 1.44, 95% confidence interval (CI) 1.02–2.02, P = 0.039). The stratified analysis suggested that increased risk associated with ATM rs189037 AA genotype in individuals who never or seldom were exposed to cooking oil fumes (adjusted OR 1.89, 95%CI 1.03–3.49, P = 0.040).

**Conclusions:**

ATM rs189037 might be associated with the risk of lung adenocarcinoma in Chinese non-smoking females. Furthermore, ATM rs189037 AA genotype might be a risk factor of lung adenocarcinoma among female non-smokers without cooking oil fume exposure.

## Introduction

Lung cancer is the leading cause of cancer-related deaths, both worldwide and in China. Although cigarette smoke is the major risk factor for lung cancer, only a fraction of smokers develop this disease [1], suggesting that host genetic susceptibility may play an important role in the development of lung cancer. Recent genetic susceptibility studies of lung cancer have focused on single nucleotide polymorphisms (SNPs) in candidate genes, among which DNA repair genes are increasingly studied because of their critical role in maintain genome integrity. Genetic variations in DNA repair genes are thought to affect DNA repair capacity and deficits in DNA repair capacity may lead to genetic instability and carcinogenesis [2], [3].

As one of the DNA repair genes, ataxia-telangiectasia mutated (ATM) gene which is responsible for the multisystem autoxomal recessive disorder ataxia-telangiectasia (A–T), plays a crucial role in the recognition, signaling and repair of DNA damage, especially DNA double-strand breaks (DSBs) [4], [5]. The ATM protein is a member of phosphoinositide 3-kinase (PI-3 kinases) and can be activated by DSBs caused by ionizing radiation or reactive oxygen intermediates [6], [7]. Once activated, ATM can phosphorylate various downstream substates that function in cell cycle arrest, apoptosis and DNA repair, such as p53, NBS1, BRCA1 and Chk2 [8], [9]. Therefore, genetic variants in ATM gene may lead to the structure and function change of the protein and act as important factors indicating individual susceptibility to cancer. ATM -111G>A (rs189037) resides in the promoter of ATM gene. Increasing studies have shown that variations in the DNA promoter sequence may potentially alter the affinities of multiple regulatory proteins-DNA interactions or the specificity of the transcriptional process [10]–[13]. Although this polymorphism makes no amino acid change, the alleles may have different binding affinity to the transcription factor and exhibit different levels of mRNA expression [14], [15]. Zhang et al. [16]declared that ATM rs189037 AA genotype was associated with a lower ATM mRNA levels than GG genotype in lung tissue samples. Their results showed that the G-to-A change might create a transcriptional inhibitor-binding site for ATM rs189037 A allele promoter and subsequently reduce the ATM mRNA expression. Consequently, lower expression of ATM might cause elevated sensitivity to ionizing radiation, defects in the activation of cell cycle checkpoints, a reduced capacity for DNA repair and abnormal apoptosis. All of these features would contribute to increased individual cancer susceptibility. In recent years, a number of studies have evaluated the association between this polymorphism and cancer risk, such as thyroid carcinoma [17], oral cancer [18], breast cancer [19], leukemia [20], nasopharyngeal carcinoma [21], glioma [22] and lung caner [23]–[25].

Previous studies of ATM rs189037 have included cigarette smokers as cases and controls that made it difficult to judge whether this polymorphism were associated with lung cancer or tobacco use. Considering the facts in China, the incidence and death rate of lung cancer in women continues to increase and this phenomenon is frequently occurring in those who have never smoked. In order to have a better control of confounding of gender or smoking, we performed a case-control study to identify the association between the polymorphism of ATM rs189037 and the risk of lung cancer in the non-smoking females in Chinese Han population. We also investigated the interaction between genetic polymorphism and environmental exposure in lung cancer.

## Methods

### Subjects

This hospital-based case-control study included 487 lung cancer patients and 516 cancer-free hospital controls. All subjects were female non-smokers and they were from unrelated ethic Han Chinese. The cases were recruited during January 2002 to November 2012 at Liaoning Cancer Hospital & Institute. All patients were histologically confirmed to have lung cancer before any radiotherapy and chemotherapy. During the same time, controls were selected from patients with other lung diseases but free of cancer history and symptom. Controls suffered mainly from bronchitis, pneumonias, fibrosis, sarcoidosis, chronic obstructive pulmonary disease and emphysema. Controls were all non-smoking females and frequency-matched to case subjects for age (±5 years). This study was approved by the institutional review board of China Medical University and written informed consent was obtained from each participant or each participant's representatives if direct consent could not be obtained.

### Data Collection

A total of 10 ml of venous blood was collected from each patient. Patients were interviewed to collect information for demographics and environmental exposure at the time they were admitted to hospital. Information concerning demographic characteristics, passive smoking, cooking oil fume exposure, fuel smoke exposure, family history of cancer, occupational exposure and dietary habit was obtained for each case and control by trained interviewers. An individual was defined as a smoker if she had consumed a total of 100 cigarettes in her lifetime; otherwise, she was considered as a non-smoker. About fuel smoke exposure, participants who used coal-fuel-burning stoves without chimneys were regarded as fuel smoke exposure. For exposure to cooking oil fumes, participants were mainly asked about the method of cooking and eyes or throat irritation. For cooking methods, participants were asked whether they cooked food in a stir-frying way and how many times a week; for eyes or throat irritation, participants were asked how often they felt eyes or throat irritated by the oily smoke. There were four possible responses ranging from “never”, “seldom”, “sometimes”, and “frequently”. Subjects were considered as cooking oil fume exposure if they met criteria as follows: (1) have cooked for over 15 years; (2) cooked food in a stir-frying way for more than twice a week; (3) felt eyes or throat irritated by oily smoke. Exposure for cooking oil fume was categorized as an indicator variable equal to 1 if participants reported frequently or sometimes, and equal to 0 otherwise.

### Genotype Analysis

Genomic DNA was extracted from peripheral blood samples by the conventional phenol-chloroform extraction method. SNP was genotyped by investigators blinded to case-control status in order to avoid any genotyping bias, using TaqMan methodology and read with the Sequence Detection Software on an Applied Biosystems 7500 FAST Real-Time PCR System according to the manufacturer's instructions (Applied Biosystems, Foster City, CA). Amplification was done under the following conditions: 95°C for 10 min followed by 47 cycles of 92°C for 30 s and 60°C for 1 min. In this study, 487 lung cancer patients and 516 controls were all genotyped successfully and 5% duplicated samples were randomly selected to assess the reproducibility for quality control, with a concordance rate of 100%.

### Statistical Analysis

The *x* ^2^ test and *t* test were applied to estimate differences in demographic variables and distributions of genotypes between cases and controls. The association of genotypes of ATM rs189037 with risk of lung cancer was estimated by computing the odds ratios (ORs) and 95% confidence intervals (CIs) in unconditional logistic regression analysis. The Hardy-Weinberg equilibrium (HWE) was tested using goodness-fit *x* ^2^ test to compare the genotype frequencies in the control subjects from those expected. A logistic regression model was used to evaluate gene-environment interactions. All data were analyzed with Statistical Product and Service Solutions (SPSS) v13.0 for Windows, if not otherwise specified. All statistical analysis were two-sided and the significance level was set at P<0.05.

## Results

### Population characteristics

A total of 487 lung cancer and 516 age-matched cancer-free controls were enrolled in this study. As shown in Table 1, the mean ages of cases and controls (mean ±S.D.) were almost identical (56.5±11.7 and 56.3±12.5, respectively). All cases were female non-smoking lung cancer patients. No statistically significant difference was found between cases and controls in terms of age (P = 0.248) and monthly income (P = 0.084). Cases included 434 non-small cell lung cancer (NSCLC) patients and 53 small cell carcinoma patients. In the NSCLC cases, there were 320 adenocarcinomas, 73 squamous cell carcinomas and 41 other tumors with a variety of different pathologies (such as large cell carcinomas, mixed cell carcinomas or undifferentiated carcinomas).

**Table 1:** Characteristics of lung cancer cases and controls.

Variables	Cases(%)	Controls(%)	*P* value
Female	487	516	
Mean age (years)	56.5±11.7	56.3±12.5	0.248^a^
Income (yuan/month)	628.9±419.3	563.5±387.6	0.084^a^
Never smoker	487	516	
Histological type			
NSCLC	434(89.1)		
Adenocarcinoma	320(65.7)		
Squamous cell carcinoma	73(15.0)		
Small cell carcinoma	53(10.9)		
Other	41(8.4)		

a Student's t-test was used to compare the frequency distributions of demographic variables between the cases and controls.

### Association analysis

The observed genotype frequencies among the control subjects was in agreement with that expected under the Hardy-Weinberg equilibrium (P = 0.119). The distribution of ATM rs189037 genotypes among subjects were displayed in Table 2. Using subjects with the ATM rs189037 GG genotype as the reference group, we calculated the ORs and 95%CIs for heterozygous carriers of GA genotype and homozygous carriers of AA genotype. No significant difference was observed between lung cancer cases and controls in each test (P>0.05). In order to increase the statistical power, we combined the GA genotype with the AA genotype to compare with GG genotype as a dominant model and combined the GA genotype with the GG genotype to compare with AA genotype as a recessive model. The results indicated that individuals with AA genotype had a significantly elevated risk of lung adenocarcinoma compared with those carrying the GG or GA genotype (OR = 1.44, 95%CI 1.02–2.02, P = 0.039).

**Table 2:** Distribution of ATM rs189037 genotypes and ORs for lung cancer cases and controls.

Genotype	Cases(%)	Controls(%)	OR^c^	95%CI	*P*
overall (n = 487)					
GG	148(30.4)	152(29.5)	ref		
GA	240(49.3)	272(52.7)	0.91	0.68–1.20	0.494
AA	99(20.3)	92(17.8)	1.11	0.77–1.59	0.590
dominant model^a^			0.96	0.73–1.25	0.742
recessive model^b^			1.18	0.86–1.61	0.313
NSCLC (n = 434)					
GG	129(29.7)	152(29.5)	ref		
GA	213(49.1)	272(52.7)	0.92	0.68–1.24	0.573
AA	92(21.2)	92(17.8)	1.18	0.81–1.71	0.397
dominant model			0.98	0.74–1.30	0.906
recessive model			1.24	0.90–1.71	0.192
Adenocarcinoma (n = 320)					
GG	94(29.4)	152(29.5)	ref		
GA	150(46.9)	272(52.7)	0.89	0.64–1.23	0.485
AA	76(23.7)	92(17.8)	1.33	0.90–1.99	0.156
dominant model			1.00	0.74–1.36	0.987
recessive model			1.44	1.02–2.02	0.039*
Squamous cell carcinoma (n = 73)					
GG	24(32.9)	152(29.5)	ref		
GA	39(53.4)	272(52.7)	0.90	0.52–1.56	0.706
AA	10(13.7)	92(17.8)	0.69	0.32–1.51	0.355
dominant model			0.85	0.50–1.43	0.537
recessive model			0.74	0.37–1.50	0.400

*P<0.05.

a GA+AA vs GG.

b AA vs GA+GG.

c adjusted for age and data were calculated by unconditional logistic regression.

According to the results above, we assumed that ATM rs189037 AA genotype might affect lung adenocarcinoma risk among non-smoking Chinese females. To test this hypothesis and explore the gene-environment interaction, we adopted all the lung adenocarcinoma patients and cancer-free controls whose information about environmental risk factors were completely obtained such as fuel smoke exposure, cooking oil fume exposure, passive smoking and family history of cancer. Cases and controls were not included in the association analysis if any item of their environmental risk factors data was incomplete. After screening, we had 242 lung adenocarcinoma cases and 277 cancer-free controls that were eligible. Selected demographic variables and environmental risk factors for the cases and controls were listed in Table 3.

**Table 3:** Basic demographic data and environmental risk factor in lung adenocarcinoma cases and controls.

Variable	Cases(%)	Controls(%)	*P* value
Female	242	277	
Mean age (±S.D.)	55.7±11.6	56.6±11.0	0.346^a^
Income(yuan/month)	626.5±384.0	558.1±391.4	0.066^a^
Education			
Never	26(10.7)	26(9.4)	0.305
Elementary school	111(45.9)	141(50.9)	
Junior school	76(31.4)	69(24.9)	
Senior school and upwards	29(12.0)	41(14.8)	
Fuel smoke exposure	66(27.3)	76(27.4)	0.967^b^
Cooking oil fume exposure	86(35.5)	70(25.3)	0.011^b^ *
Family history of cancer	26(10.7)	30(10.8)	0.975^b^
Passive smoking exposure	141(58.3)	158(57.0)	0.778^b^

^*^P<0.05.

a Student's t-test was used to compare the frequency distribution of demographic variables between the cases and controls.

b Peason's chi square was used to compare the frequency distribution of demographic variables, fuel smoke exposure, cooking oil fume exposure, family history of cancer, passive smoking between the cases and controls.

As shown in Table 3, the mean ages of lung adenocarcinoma cases and controls (mean±S.D.) were similar (P>0.05). All cases were female non-smokers. There was no significant difference in the distribution of education, fuel smoke exposure, family history of cancer and passive smoking status between cases and controls. However the cases were more likely than the controls to report cooking oil fume exposure (P = 0.011). Table 4 summarized the relationship between ATM rs189037 genotypes and lung adenocarcinoma risk with the stratification analysis of cooking oil fume exposure. The results indicated that in the recessive model (AA vs GA/GG), individuals carrying AA genotype had a 1.69-fold risk of lung adenocarcinoma compared with those carrying GA or AA genotype (95%CI 1.09–2.61, P = 0.019). Considering the difference in the distribution of cooking oil fume exposure between cases and controls, we conducted the stratification analysis. Our data revealed that AA homozygous carriers had an increased risk of lung adenocarcinoma among women who were never or seldom exposed to cooking oil fume (OR = 1.89, 95%CI 1.03–3.49, P = 0.040). To well-understood the possible interaction between rs189037 polymorphism and cooking oil fumes exposure, next we conducted a combined analysis. But there was no significant correlation found between this SNP and cooking oil fumes exposure.

**Table 4:** Overall association, stratification analysis and combined analysis between ATM rs189037 polymorphism and lung adenocarcinoma risk.

Comparison model	Genotype	OR^c^	95%CI	*P* value
Overall				
	GG	ref		
	GA	0.89	0.59–1.35	0.592
	AA	1.57	0.93–2.62	0.089
	dominant model^a^	1.05	0.70–1.55	0.825
	recessive model^b^	1.69	1.09–2.61	0.019*
Stratified by cooking oil fuel exposure				
Yes	GG	ref		
	GA	0.72	0.34–1.54	0.395
	AA	1.19	0.43–3.24	0.740
	dominant model^a^	0.81	0.39–1.68	0.573
	recessive model^b^	1.48	0.62–3.52	0.381
No	GG	ref		
	GA	0.94	0.57–1.56	0.811
	AA	1.89	1.03–3.49	0.040*
	dominant model^a^	1.16	0.72–1.87	0.542
	recessive model^b^	1.97	1.18–3.29	0.009*
Cooking oil fumes exposure-genotype combined analysis				
non-exposed	GG	ref		
non-exposed	GA	0.91	0.55–1.50	0.714
non-exposed	AA	1.71	0.94–3.09	0.078
exposed	GG	1.99	0.96–4.12	0.063
exposed	GA	1.58	0.88–2.82	0.127
exposed	AA	2.07	0.87–4.93	0.099

*P<0.05.

a GA+AA vs GG.

b AA vs GA+GG.

c the overall test was adjusted for age, fuel smoke exposure, cooking oil fume exposure, family history of cancer and passive smoking and the stratified analysis was adjusted for age, fuel smoke exposure, family history of cancer and passive smoking.

## Discussion

It is well known that smoking is the most important risk factor for lung cancer, but in the past 30years, the incidence and death rate of lung cancer continues to increase in women who have a low rate of smoking [26]–[28]. Adenocarcinoma accounts for about 40% of all lung cancer, with a higher incidence in women, especially in those who have never smoked. Undoubtedly female non-smokers are the ideal subjects to examine unknown, yet important environmental and genetic factors of lung adenocarcinoma. Exposure to cooking oil fume, fuel smoke, passive smoking and occupational exposures have been implicated as possible risk factors among Chinese women mainly based on Chinese people's traditional diet habits, lifestyle and social environment [29], [30]. So we designed this study to evaluate the association between genetic variant and environmental risk factors and lung adenocarcinoma in female non-smokers. To date, the association between ATM rs189037 and host susceptibility to lung adenocarcinoma in Chinese female non-smokers has not been well addressed.

ATM rs189037 was a common polymorphism in the promoter of ATM gene. Studies have shown that this site possibly may regulate ATM protein activity due to regulation function of promoter as shown in most genes. And specific genotypes or haplotypes of ATM may play an important role in carcinogenesis through expression regulation or alternative splicing of the ATM gene [31]. We searched through NCBI (National Center for Biotechnology Information) dbSNP database to get the allele frequency of this polymorphism. The data indicated that the frequency of wild-type allele G was 61.1% and the frequency of variant allele A was 38.9% in Chinese Han population (http://www.ncbi.nlm.nih.gov/SNP/snp_ref.cgi?rs=rs189037). In this study, our results was in accordance with the data from NCBI. In 2006, Kim et al.[25] evaluated the role of ATM rs189037 in lung cancer development In Korean population for the first time. No significant association was found between this polymorphism and lung cancer risk (P>0.05). They recruited 616 lung cancer patients, in which 78.4% were male and 79.6% were cigarette smokers. As cigarette smoking might modulate the risk of lung cancer, in turn it could be a confounder in the association between ATM rs189037 and lung cancer risk. Besides, there was no gene-environment interactions be considered in their research. In 2010, Lo et al.[23] suggested that ATM rs189037 was associated with lung cancer risk among never smokers (AA vs GG: OR = 1.61, 95%CI 1.10–2.35) and this association might be modified by passive smoking. Although they have eliminated the cofounding effect of cigarette smoking by conducting their study in non-smokers, the risk of lung cancer among different histological types still needed to be clarified. Recently, Hsia et al.[24] put their attention on the association of ATM rs189037 with lung cancer susceptibility among ever smokers. No genotype frequency difference was found between lung cancer cases and controls among ever smokers (P>0.05). After summing up the omissions of their studies and combining with the current situation that Chinese non-smoking female lung adenocarcinoma incidence and fatality rate was increasingly rising up, we performed this case-control study to elucidate the association between ATM rs189037 and lung adenocarcinoma risk. To the best of our knowledge, this is the first study that has investigated whether ATM rs189037 was associated with lung adenocarcinoma risk in non-smoking Han-Chinese females.

Our results have shown that individuals with exposure to cooking oil fume had a 1.63-fold increased risk of developing lung adenocarcinoma (P = 0.011). Similar significant associations were observed in our previous studies of Chinese non-smoking females. Experimental studies have presented that fumes from cooking oils could be genotoxic because of the potential carcinogenic components such as polycyclic aromatic hydrocarbons (PAHs) and benzo[a]pyrene 7,8-diol 9,10-epoxide (BPDE), which involved in inducing DNA adducts and thus made a predisposition to lung adenocarcinoma [32]–[34]. Besides the method of cooking and throat or eyes irritation, the interviewers also asked each woman the information on cooking oil fumes exposure such as the types of cooking oils she used, the frequency she used stir frying or deep frying to prepare food, ventilation conditions and the use of a fume extractor. Increasing epidemiological studies have reported cooking method and types of cooking oils on lung cancer susceptibility among Chinese females. Seow et al.[35]found that women who reported that they stir fried daily had a significantly increased risk of lung cancer (OR = 2.0, 95%CI 1.0–3.8) and risk was enhanced for those who stir fried meat daily (OR = 2.7, 95%CI 1.3–5.5). The elevated lung cancer risk might be attributed to heterocyclic amines generated during frying of meats. In addition, the frequency of stir frying seemed to be related with lung cancer susceptibility. Gao et al. [36]investigated the association between the frequency of stir frying and lung cancer risk in Chinese females, they observed that stir frying more than 30 dishes per week was associated with high risk of lung cancer (OR = 2.6, 95%CI 1.3–5.0). In a case-control study in northeast China, Wu-Williams et al.[37] found that women who deep fried twice per month had a 2.1-fold increased risk of developing lung cancer than those who never used deep frying method. And there was a significant trend in risk with increasing number of meals cooked by deep frying. Also this kind of correlation was found in both non-smokers and lung adenocarcinoma population. For types of cooking oils, Zhong et al.[38] reported that soybean oil was most commonly used in Shanghai and the use of rapeseed oil was associated with a higher risk of lung cancer (OR = 1.84, 95%CI 1.12–3.03).

In this study, we observed that ATM rs189037 AA genotype carriers were more susceptible to lung adenocarcinoma than GA or GG genotype carriers in a recessive model. This might not give direct support for AA genotype as a risk factor for lung adenocarcinoma. But the results reflected that G allele might be a protective factor for lung adenocarcinoma. So we compared AA genotype with GA genotype and our data showed that women who were AA genotype carriers had an elevated risk of lung adenocarcinoma (OR = 1.74, 95%CI 1.10–2.74, P = 0.018). In other words, GA genotype might be protective for developing lung adenocarcinoma. In the stratified analysis of cooking oil fumes exposure, we also found that AA genotype carriers had a predisposition to lung adenocarcinoma in women who had no exposure of cooking oil fumes (OR = 1.89, 95%CI 1.03–3.49). Considering that G allele might be a protective factor for lung adenocarcinoma, we then compared AA genotype with GA genotype to further validate our previous results. And it turned out that in the non-exposed group women who were AA genotype carriers had a higher risk of lung adenocarcinoma than those GA genotype carriers (OR = 1.98, 95%CI 1.15–3.40, P = 0.014), which was in accordance with our previous data that G allele might be a protective factor for lung adenocarcinoma. But in the combined analysis of interaction of cooking oil fumes exposure and rs189037 polymorphism, no significant association was found. We have described the distribution of any possible factors such as age, passive smoking status, fuel smoke exposure, family history of cancer between cooking oil fumes exposed group and non-exposed group that might affect the association, but none of these seemed to be different between exposed group and non-exposed group (Table 5). As tumor is a multifactorial disease, we could infer that there might be other risk factors playing a role in the development of lung adenocarcinoma. We tended to believe that there might be other host genetic susceptibility or unknown risk factors caused the results.

**Table 5:** Comparisons of distribution of risk factors between cooking oil fumes exposed group and non-exposed group.

Variable	Exposed(%)	Non-exposed(%)	*P* value
Mean age (±S.D.)	56.3±11.7	56.1±11.1	0.871^a^
Fuel smoke exposure	44(28.2%)	98(27.0%)	0.777^b^
Passive smoking exposure	96(61.5%)	203(55.9%)	0.235^b^
Family history of cancer	19(12.2%)	37(10.2%)	0.504^b^

a Student's t-test was used to compare the frequency distribution of demographic variables between the exposed group and non-exposed group.

b Peason's chi square was used to compare the frequency distribution of demographic variables, fuel smoke exposure, family history of cancer, passive smoking between the exposed group and non-exposed group.

There are several limitations in the current study. First, hospital-based studies are likely to include some controls with non-malignant lung diseases, especially those associated with chronic inflammatory processes, are suspected to have predisposing factors for lung cancer. The ORs we found may be underestimated. Second, the statistical power of the study may be limited by the relatively small sample size of subjects. In addition, other SNPs in ATM gene and in this pathway may be involved in the risk of lung adenocarcinoma, gene-gene interaction and haplotypes may offer more clues to clarity the association between host genetic susceptibility and lung adenocarcinoma risk.

But it is noteworthy that our study investigated the association between ATM rs189037 polymorphism and lung adenocarcinoma risk in a non-smoking females population for the first time. Meanwhile we explored the combined effects of cooking oil fumes exposure and ATM rs189037 polymorphism on lung adenocarcinoma risk. As our small sample size and only one SNP genotyped, large-scale studies with gene-gene and gene-environment interactions in different races and population are required to validate our findings.

## Conclusions

In summary, this hospital-based case-control study showed that ATM rs189037 might be associated with the risk of lung adenocarcinoma in Chinese non-smoking females. Furthermore, ATM rs189037 AA genotype might be a risk factor affecting lung adenocarcinoma among females without cooking oil fume exposure.
